# The Association of Maximum Body Weight on the Development of Type 2 Diabetes and Microvascular Complications: MAXWEL Study

**DOI:** 10.1371/journal.pone.0080525

**Published:** 2013-12-04

**Authors:** Soo Lim, Kyoung Min Kim, Min Joo Kim, Se Joon Woo, Sung Hee Choi, Kyong Soo Park, Hak Chul Jang, James B. Meigs, Deborah J. Wexler

**Affiliations:** 1 Department of Internal Medicine, Seoul National University College of Medicine and Seoul National University Bundang Hospital, Seongnam, Korea; 2 Department of Ophthalmology, Seoul National University College of Medicine and Seoul National University Bundang Hospital, Seongnam, Korea; 3 Department of Internal Medicine, Korea Cancer Center Hospital, Seoul, Korea; 4 Department of Internal Medicine, Seoul National University College of Medicine, Seoul, Korea; 5 Division of General Medicine, Harvard Medical School, Boston, Massachusetts, United States of America; 6 Diabetes Center, Harvard Medical School, Boston, Massachusetts, United States of America; 7 Massachusetts General Hospital and Harvard Medical School, Boston, Massachusetts, United States of America; University of Tolima, Colombia

## Abstract

**Background:**

Obesity precedes the development of type 2 diabetes (T2D). However, the relationship between the magnitude and rate of weight gain to T2D development and complications, especially in non-White populations, has received less attention.

**Methods and Findings:**

We determined the association of rate and magnitude of weight gain to age at T2D diagnosis (Age_T2D_), HbA1c at T2D diagnosis (HbA1c_T2D_), microalbuminuria, and diabetic retinopathy after adjusting for sex, BMI at age 20 years, lifestyles, family history of T2D and/or blood pressure and lipids in 2164 Korean subjects aged ≥30 years and newly diagnosed with diabetes. Body weight at age 20 years (Wt_20y_) was obtained by recall or from participants’ medical, school, or military records. Participants recalled their maximum weight (Wt_max_) prior to T2D diagnosis and age at maximum weight (Age_max_wt_). The rate of weight gain (Rate_max_wt_) was calculated from magnitude of weight gain (ΔWt = Wt_max_–Wt_20y_) divided by ΔTime (Age_max_wt_ –20 years). The mean Age_max_wt_ and Age_T2D_ were 41.5±10.9 years and 50.1±10.5 years, respectively. The Wt_20y_ and Wt_max_ were 59.9±10.5 kg and 72.9±11.4 kg, respectively. The Rate_max_wt_ was 0.56±0.50 kg/year. After adjusting for risk factors, greater ΔWt and higher Rate_max_wt_ were significantly associated with earlier Age_T2D,_ higher HbA1c_T2D_ after additional adjusting for Age_T2D,_ and microalbuminuria after further adjusting for HbA1c_T2D_ and lipid profiles. Greater ΔWt and higher Rate_max_wt_ were also significantly associated with diabetic retinopathy.

**Conclusions:**

This finding supports public health recommendations to reduce the risk of T2D and its complications by preventing weight gain from early adulthood.

## Introduction

The world prevalence of diabetes among adults (aged 20–79 years) was 6.4%, affecting 285 million adults, in 2010, and will increase to 7.7%, and 439 million adults by 2030 [1]. Primary prevention of diabetes and its complications is now an important public health priority worldwide [2].

Obesity is the major risk factor for developing type 2 diabetes mellitus (T2D) [3]. Obesity increases insulin resistance in tissues such as muscle, liver, and adipose tissue. In response to this condition, the pancreatic beta-cells increase insulin production to decrease blood glucose level. Thus, obesity has direct connection with insulin resistance; a condition characterized by increased insulin production and impaired glucose tolerance [4]. Many studies have reported associations between body mass index (BMI) and T2D [5]–[8]. These studies have shown that besides obesity *per se*, an increase in body weight of 3–20 kg is associated with an elevated risk of incidence of T2D. Prevention of weight gain is beneficial for the prevention of T2D in many different ethnicities [9]–[11].

While obesity antedates the development of T2D by some years, quantitative investigation of the relationship between magnitude and rate of weight gain and the development of T2D has been relatively limited, especially in non-White populations. The present study was designed to examine the association of development of T2D and glycemia at diagnosis with weight at age 20 years, maximum lifetime weight before T2D diagnosis, age at maximum weight, and the rate of weight gain, and to identify which of these variables were most predictive of development of T2D, glucose control, and microvascular complications such as microalbuminuria and diabetic nephropathy. We hypothesized that rapid and greater weight gain would increase the risk of T2D diagnosis and its complications.

## Methods

### Study Population

The MAXWEL cohort was established in 2006 to investigate the effect of maximum body weight and time interval to maximum body weight on the development of T2D. We consecutively screened all individuals (n = 5,321) aged over 30 years who visited the diabetes clinic first for initial diabetes evaluation at Seoul National University Bundang Hospital (SNUBH), Seongnam, Korea, from January 2007 to December 2009.

After excluding previously diagnosed cases with diabetes, we selected 2977 subjects who had confirmed T2D by glycosylated hemoglobin (HbA1c) ≥6.5%, based on the American Diabetes Association diagnosis criteria for diabetes [12], and not on antidiabetic medications for more than 1 week before. Of these, those with type 1 diabetes (measured by Glutamic Acid Decarboxylase antibody, n = 32), gestational diabetes (n = 12), or diabetes with secondary causes (n = 16). Patients with malignancy (n = 44), chronic obstructive pulmonary disease (n = 68), depression and/or eating disorder (n = 39), chronic gastrointestinal disorders (n = 39), any medication for weight control for more than 3 months (n = 37), and organ transplantation (n = 4) were excluded. Another 522 subjects were excluded because they were not able to recall their maximum weight or age at maximum weight. They were similar to other participants in anthropometric and biochemical parameters such as age, sex, and glucose control. A total of 2164 newly detected T2D subjects (1220 men and 944 women) men from 2007 to 2009 were included in the current analysis. Medical history and biochemical tests including fasting glucose, HbA1c, and lipid profiles were obtained at the first visit.

The protocol was reviewed and approved by the institutional review board (IRB) of SNUBH (No. B-0909/083-008) and the patient informed consent requirement was waived by the IRB.

### Assessment of Weight-related Information

**Figure 1:**
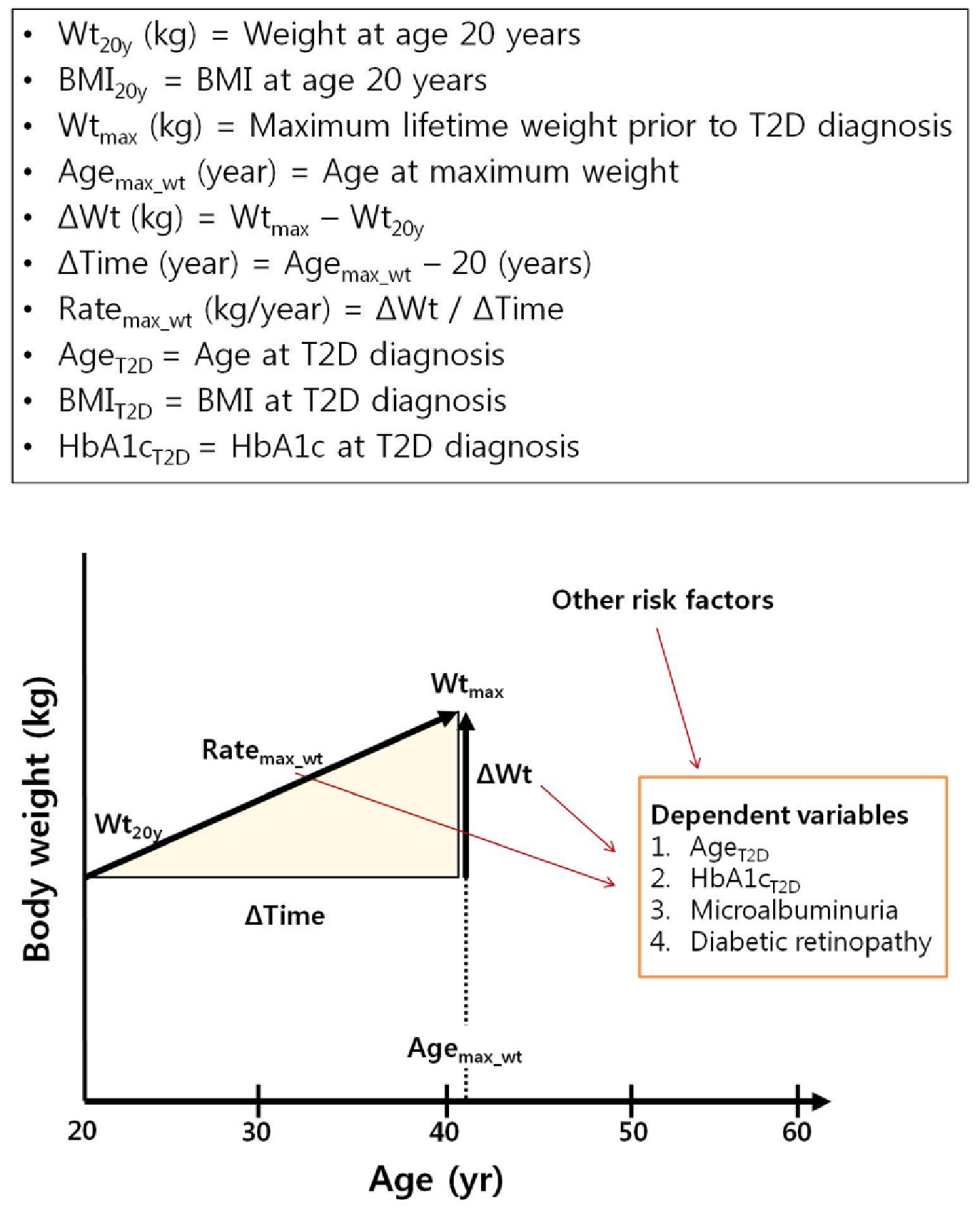
Definitions of weight-related variables and study design.

Body weight at age 20 years (Wt_20y_) was obtained in 94.5% of study subjects from the following sources: medical records, military service or college examination records, or personal recording. The remaining 5.5% self-reported their Wt_20y_. Maximum weight before T2D diagnosis (Wt_max_) and age at maximum weight (Age_max_wt_) were also self-reported. Weight around pregnancies was disregarded. In 31.3% subjects who were randomly selected from all participants (n = 678), the recalled Wt_max_ was validated by written document, and the agreement rate was high (r = 0.91). We calculated the rate of weight gain (Rate_max_wt_), which was defined as the slope, where weight change (in kilograms) from age 20 years to maximum weight was divided by the time between age 20 years and age at maximum weight (in years). Definition of weight-related variables and study design were shown in **Figure 1**.

### Assessment of Lifestyle and Characteristics

Interviews were conducted by designated physicians using a standardized survey querying smoking status, alcohol consumption, and exercise habits. Smoking status was divided into three categories: current smokers, ex-smokers and never smokers. Alcohol intake was assessed by frequency and quantity of beer, spirit, sake, and wine intake during the last 12 months. Alcohol intake in grams of alcohol per week was categorized into two categories: ≤ moderate (≤199.9 g/week) and heavy intake (≥200 g/week). Physical activity was classified into three categories: no, irregular (≤2/wk) and regular (≥3/wk) exercise. One episode of exercise was defined as exercising for at least 30 min.

### Anthropometric and Biochemical Parameters

Height and body weight were measured at the time of T2D diagnosis by standard methods. BMI was calculated as body weight divided by the height squared (kg/m^2^). Blood pressure measurements were made after subjects had remained seated for 10 min. Measurements were made twice, with a 5-min rest period; the mean value of measurements was used.

We measured HbA1c for diabetes diagnosis along with fasting glucose and insulin and other biochemical parameters in a 12-h fasting state. HbA1c was measured by Bio-Rad Variant II Turbo HPLC analyzer (Bio-Rad, Hercules, CA, USA) in SNUBH, the National Glycohemoglobin Standardization Program (NGSP) level II certified laboratory. The fasting plasma concentrations of glucose, total cholesterol, triglyceride, and high-density lipoprotein (HDL) cholesterol were measured using the Hitachi 747 chemistry analyzer (Hitachi, Tokyo, Japan). Fasting plasma insulin concentrations were measured by radioimmunoassay (Linco, St. Louis, MO, USA).

### Microalbuminuria

Urinary albumin levels were measured by turbitimer assay (A&T 502X, A&T, Tokyo) and urine creatinine levels were measured by the Jaffe method (Hitachi 7170, Hitachi, Tokyo) to calculate spot urine albumin-to-creatinine ratio (U_ACR_). Microalbuminuria was defined by U_ACR_ ≥30 (mg/g).

### Diabetic Retinopathy

Complete ophthalmologic examinations including funduscopy on the entire retina after mydriasis were performed on all patients by two ophthalmologists. After the thorough funduscopic examination, patients showing any features of diabetic retinopathy underwent color fundus photography using mydriatic 45° fundus camera (VX-10α, Kowa Inc., Nagoya, Japan). The presence and severity of diabetic retinopathy were graded based on international clinical diabetic retinopathy severity scales proposed by the Global Diabetic Retinopathy Project Group [13]. Non-proliferative diabetic retinopathy (NPDR) was defined as the presence of at least one definite retinal hemorrhage and/or microaneurysm. Subjects were assigned to the PDR group when retinal neovascularization was visible on retinal photographs.

### Statistical Analysis

All data are presented as the mean and SD, and were analyzed using SPSS for Windows version 17.0 (SPSS Inc., Chicago, IL, USA). The distributions of triglycerides and U_ACR_ were skewed (Kolmogorov-Smirnov Z = 1.22 and Z = 1.19, both *P*<0.05). Those values were normalized by logarithmic transformation for all analyses. The variables were compared using student’s *t* or χ^2^ tests. Correlations between variables were analyzed using Pearson’s correlation.

We compared mean values of Age_T2D_, HbA1c_T2D_ and microalbuminuria, and prevalence of diabetic retinopathy between the highest and lowest quartile of Rate_max_wt_.

To test independent association of weight variables, we performed three multivariable linear regression models for Age_T2D_, HbA1c_T2D_ and U_ACR_, respectively, and one multivariable logistic regression model for diabetic retinopathy.

For Age_T2D_, ΔWt and Rate_max_wt_ were included as key independent variables in the multivariable linear regression model with sex, BMI_20y_, alcohol consumption, smoking status, exercise habits and family history of diabetes as covariates. In the multivariable linear regression analysis for HbA1c_T2D_, Age_T2D_ was additionally added as a covariate because glycaemic control might be influenced by age of diagnosis. For log-transformed U_ACR_, Age_T2D_, systolic blood pressure (SBP), diastolic blood pressure (DBP), HbA1c_T2D_, and log-transformed triglycerides/HDL-cholesterol ratio were additionally added to the multivariable linear regression model because these variables might be able to affect kidney function. To assess multicollinearity of the linear regression models, we checked the variance inflation factor of variables.

For diabetic retinopathy (combined NPDR and PDR), a multivariable logistic regression analysis was performed with the same variables used in the model for U_ACR;_ SBP≥140 mmHg or blood pressure medication indicated hypertension to obtain an odds ratio. Since multiple tests were performed in the analysis, we adjusted the number (n = 4) of phenotypes, by multiplying *P* values by 4. These significance thresholds are conservative given correlation among the phenotype traits themselves. Statistical significance was defined as *P*<0.05.

## Results

### Baseline Characteristics of the Participants

The baseline characteristics of the 2164 participants are shown in **Table 1**. The ranges of Age_T2D_ and BMI_T2D_ were 30–75 years and 15.4–40.1 kg/m^2^, respectively. Almost half of participants had a family history of diabetes. About one fourth of participants (24.3%) had microalbuminuria defined by ≥30 of U_ACR,_ and one eighth of participants (12.4%) had diabetic retinopathy at the time of T2D diagnosis.

**Table 1:** Anthropometric and biochemical parameters at T2D diagnosis and weight related variables*.

		Mean		SD
Female (%)		43.6%		
Age_T2D_ (years)		50.1		10.5
Height (cm)		163.3		8.8
Weight (kg)		68.0		11.9
BMI (kg/m^2^)		25.4		3.7
SBP (mmHg)		130.3		15.8
DBP (mmHg)		78.4		10.8
Total cholesterol (mg/dl)		202.0		39.5
Triglycerides (mg/dl)		159.4		92.9
HDL-cholesterol (mg/dl)		51.2		13.4
Fasting plasma glucose (mg/dl)		107.6		29.1
Fasting plasma insulin (µIU/ml)		153.2		6343
HbA1c_T2D_ (%)		8.0		1.5
U_ACR_ (urine albumin-to-creatinine, mg/g Cr)		72.8		335.7
Family history of diabetes				47.4%
Smoking status	*Non*			60.9%
	*Ex*			13.1%
	*Current*			26.0%
Alcohol consumption	*≤ Moderate*			86.4%
	*Heavy*			13.6%
Exercise habits	*No*			24.4%
	*Irregular*			42.5%
	*Regular*			33.1%
Diabetic retinopathy	*Normal*			87.6%
	*Nonproliferative diabetic retinopathy*			9.0%
	*Proliferative diabetic retinopathy*			3.4%
***Weight related variables***				
BMI_20y_ (BMI at age 20 year, kg/m^2^)			22.4	3.1
Wt_max_ (Maximum weight, kg)			72.9	11.4
Wt_20y_ (weight at age 20 years, kg)			59.9	10.5
ΔWt (Wt_max_ – Wt_20y_)			13.0	8.5
Age_max_wt_ (Age at maximum weight, years)			41.5	10.9
ΔTime (Age_max_wt_ –20 years)			21.5	10.9
Rate_max_wt_ (ΔWt/ΔTime)			0.56	0.50

* Data are mean and SD or percent.

### Weight-related Variables

Weight at age 20 years was 59.9 and maximum lifetime weight was 72.9 kg, resulting in 13.0 kg of change in body weight from age 20 years to maximum weight (ΔWt) (**Table 1**). Age at maximum weight (Age_max_wt_) was 41.5 years and accordingly it was 21.5 years from age 20 years to Age_max_wt_ before T2D diagnosis (ΔTime). From these two variables, the Rate_max_wt_ was calculated to be 0.56 kg/year. Seventy four subjects (3.4%) of all participants reported weight loss since age 30 years. In comparison between genders, men showed greater and more rapid weight gain than women.

### Association among Age_T2D_, ΔWt, and ΔTime

**Figure 2:**
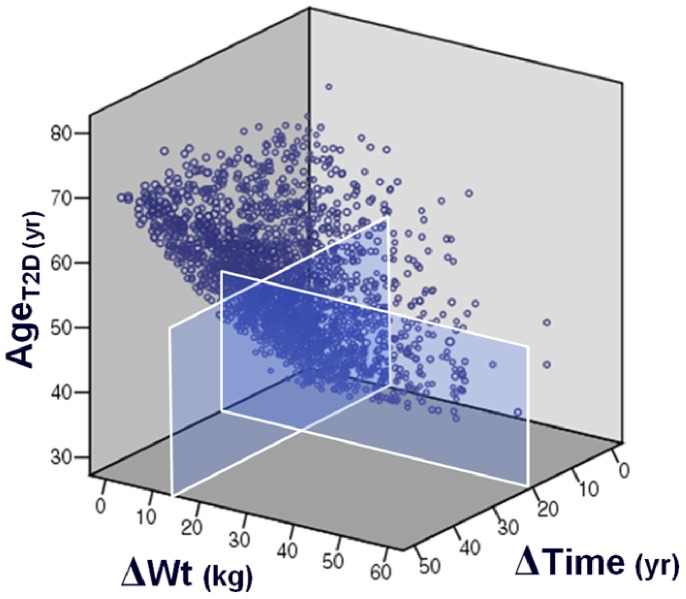
Three-dimensional graphs showing the association among three factors: Age_T2D_ (age at diagnosis of type 2 diabetes), ΔWt (maximum weight in lifetime – weight at age 20 years), and ΔTime (age at maximum weight –20 years).

**Figure 2** shows a three-dimensional graph illustrating association among Age_T2D_, ΔWt, and ΔTime without the participants who lost weight (n = 74). The Age_T2D_ decreased as ΔWt increased and as ΔTime decreased (Pearson’s correlation coefficients were –0.220 between Age_T2D_ and ΔWt and 0.495 between Age_T2D_ and ΔTime, both *P*<0.01). This illustrates subjects with greater weight gain and shorter duration to maximum weight showed a tendency to be diagnosed with T2D earlier.

### Comparison between Rapid and Slow Weight Gainers

**Figure 3:**
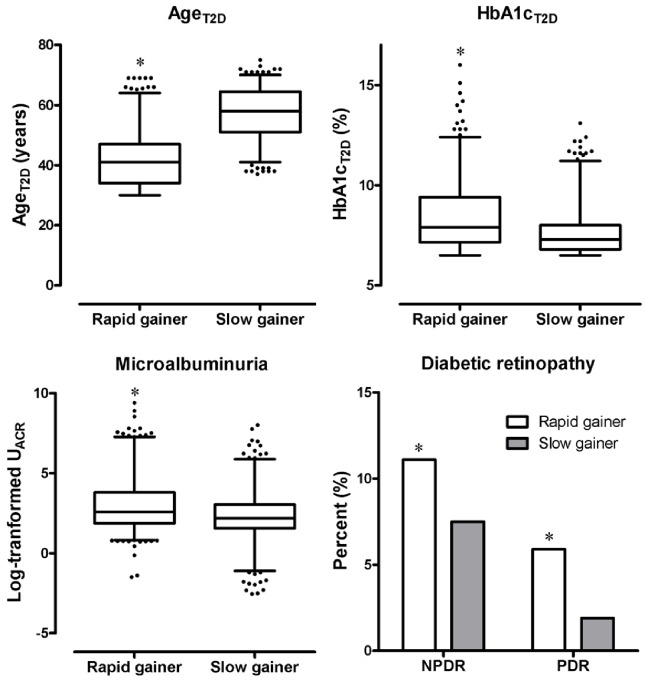
Comparison of age at T2D diagnosis, HbA1c at T2D diagnosis, microalbuminuria, and diabetic retinopathy between rapid and slow weight gainers defined as the highest and lowest quartiles of Rate_max_wt_. Mean values with 25–75% ranges in box and 5–95% ranges in outer lines are displayed in Box and Whisker plots. Outliers who do not belong to the 5–95% ranges are displayed as dots. Log-transformed urine albumin-to-creatinine ratio was used for microalbuminuria. *indicates *P*<0.01.

After excluding 74 participants who lost weight, we compared Age_T2D_, HbA1c_T2D_, microalbuminuria, and diabetic retinopathy (**Figure 3**) between the highest (4.51±9.84, n = 531) and lowest (0.14±0.09, n = 534) quartiles of Rate_max_wt_. The rapid weight gainers showed earlier T2D diagnosis (Age_T2D_), higher HbA1c level at diagnosis (HbA1c_T2D_), and greater log-transformed U_ACR_ than those of lower weight gainers (42.1±9.2 years vs. 57.3±8.6 years, 8.5±1.7% vs. 7.6±1.1%, and 3.0±1.6 vs. 2.3±1.6, respectively, all *P*<0.01). The prevalence of diabetic retinopathy was also higher in rapid compared to slow weight gainers.

### Association with Age_T2D_

In the multivariable linear regression for Age_T2D_ (**Table 2a**), greater BMI_20y_, heavy alcohol consumption, no exercise, positive family history of diabetes, greater ΔWt and higher Rate_max_wt_ were significantly associated with earlier Age_T2D_. When the diagnosis of T2DM was based on fasting glucose concentration (≥126 mg/dl), similar result was obtained (data not shown).

**Table 2:** Variables associated with age at T2D diagnosis, HbA1c at T2D diagnosis, and urine albumin-to-creatinine ratio (U_ACR_) at T2D diagnosis.

			StandardizedBeta			*P* *		95% CI		
								Lower		Upper
***a. For age at T2D diagnosis*** †										
BMI_20y_ (kg/m^2^)		−0.09			0.001			−0.46		−0.17
Alcohol (moderate or less vs. heavy)		−0.05			0.044			−2.82		−0.22
Smoking (non vs. ex vs. current)		−0.16			<0.001			−2.73		−1.71
Exercise (regular vs. irregular vs. no)		−0.05			0.024			−2.04		−0.22
Family history of diabetes (no vs. yes)		−0.10			<0.001			−3.12		−1.51
ΔWt (kg)		−0.22			<0.001			−0.35		−0.03
Rate_max_wt_ (kg/year)		−0.17			<0.001			−0.37		−0.03
***b. For HbA1c at T2D diagnosis*** ‡										
Age_T2D_ (year)		−0.15			<0.001		−0.04		−0.02	
Smoking (non vs. ex vs. current)		0.06			0.016		0.03		0.20	
Exercise (regular vs. irregular vs. no)		0.03			0.023		−0.04		0.22	
ΔWt (kg)		0.16			<0.001		0.02		0.04	
Rate_max_wt_ (kg/year)		0.12			<0.001		0.02		0.04	
***c. For U_ACR_*** # ***at T2D diagnosis*** ¶										
BMI_20y_ (kg/m^2^)	0.07			<0.001			0.01		0.06	
SBP (mmHg)	0.08			<0.001			0.01		0.02	
HbA1c_T2D_ (%)	0.17			<0.001			0.14		0.23	
Log-triglyceride/HDL-cholesterol	0.06			0.040			0.53		4.72	
ΔWt (kg)	0.14			<0.001			0.02		0.05	
Rate_max_wt_ (kg/year)	0.10			<0.001			0.01		0.04	

* Corrected P by Bonferroni method,

† Common covariates: sex, BMI_20y_, SBP, DBP, alcohol intake, smoking status, exercise habit, family history of diabetes, ΔWt, and Rate_max_wt_.

‡ Common covariates+Age_T2D_,

¶ Common covariates+Age_T2D_+HbA1c_T2D_+log-triglyceride/HDL-cholesterol.

# Log-transformed value was used.

### Association with HbA1c_T2D_

In the multivariable linear regression analysis additionally adjusted for Age_T2D_ (**Table 2b**), the subjects with early diagnosis of T2D diagnosis, ever smoker, no exercise, greater ΔWt, and higher rate of weight gain showed higher HbA1c level at diagnosis.

### Association with U_ACR_

We conducted another multivariable linear regression analysis for U_ACR_ with weight-related variables (**Table 2c**). In addition to covariates used in previous model, SBP, log-transformed triglycerides/HDL-cholesterol ratio, and HbA1c_T2D_ were added as covariates.

High BMI at age 20 years, high SBP, high HbA1c_T2D_, high log-triglyceride/HDL-cholesterol, greater ΔWt, and higher rate of weight gain were significantly associated with log-transformed U_ACR_ (**Table 2c**).

Variance inflation factors of all independent factors were less than 1.21, suggesting that there was no significant collinearity among the covariates in the regression models.

### Variables Associated with Diabetic Retinopathy

Using a multivariable logistic regression model, we further investigated the independent risk of weight-related variables for the concomitant diabetic retinopathy, where NPDR and PDR were combined. After adjusting for the same variables used in the model for U_ACR_, high BMI at age 20 years, high SBP or medication, high HbA1c_T2D_, greater ΔWt, and high rate of weight gain were found to be significantly associated with presence of diabetic retinopathy at the time of T2D diagnosis (**Table 3**).

**Table 3:** Variables associated with diabetic retinopathy† ‡.

	OR	95% CI		*P* *
		Lower	Upper	
BMI_20y_ (kg/m^2^)	1.07	1.01	1.13	0.002
SBP/DBP≥140/90 mmHg or blood pressure medication	2.86	2.21	4.41	<0.001
HbA1c_T2D_ (%)	1.22	1.12	1.31	<0.001
ΔWt (kg)	1.03	1.01	1.05	<0.001
Rate_max_wt_ (kg/year)	1.02	1.01	1.05	0.032

* Corrected P by Bonferroni method,

† Covariates: Age_T2D_, sex, BMI_20y_, SBP/DBP≥140/90 mmHg or blood pressure medication, alcohol intake, smoking status, exercise habit, family history of diabetes, HbA1c_T2D_, log-triglyceride/HDL-cholesterol, ΔWt, and Rate_max_wt_,

‡ Both nonproliferative and proliferative diabetic retinopathy were combined.

### Gender Difference in the Association of Weight Variables with Diabetic Complications

In gender-specific comparison, similar results were found with slight attenuation in the association of the Rate_max_wt_ with age at T2D diagnosis, HbA1c at T2D diagnosis, urine albumin-to-creatinine ratio at T2D diagnosis (**Table A in File S1** for men and **Table B in File S1** for women), and diabetic retinopathy (**Table C in File S1** for men and **Table D in File S1** for women), respectively.

## Discussion

In the MAXWEL cohort, greater and rapid weight gain were significant predictors of early diagnosis of T2D, high HbA1c level at diagnosis, and microalbuminuria independent of other important clinical variables. The magnitude and the rate of weight gain were also independently associated with diabetic retinopathy. These results quantitate the increased risk associated with magnitude and rate of weight gain, which are associated with earlier diagnosis of diabetes, poor glycemic control, and microvascular complications, independent of other common risk factors.

Previous studies mainly focused on amount of weight gain during a certain period. In the US First National Health and Nutrition Examination Survey, weight gain for 10 years was associated with substantially increased risk of diabetes among overweight adults [14]. Another study from US showed that there was a progressive rise in weight before development of diabetes [15]. More specifically, in a previous study, gain of >10% of body weight was associated with a significant increase in risk of T2D compared with stable weight after adjustment for multiple risk factors including initial BMI [7]. In another study, weight gain dose-dependently increased risk of T2D even among non-obese men with a low initial BMI <21 kg/m^2^ [16].

In contrast with previous studies, we considered time and magnitude of weight gain together. In our study, the beta coefficient of rate of weight gain for age at T2D diagnosis was −0.166, corresponding to 1 year earlier T2D diagnosis with 6 kg/year of rate in weight gain (−0.996 year = −0.166 year/kg × 6 kg).

The impact of obesity or weight gain on T2D incidence may differ depending on when obesity is assessed [16]–[18]. A previous study from Pima Indians showed that weight in childhood and adolescence was one of the most significant predictors of T2D [19]. Ford et al. found that participants who gained more than 5 kg over the previous 10 years had a higher chance of diabetes compared with participants whose weights remained relatively stable, even at overweight or obese levels, in a US national cohort [5]. A study from the UK showed that >10% weight gain was associated with a significant increase in risk of T2D compared to stable weight after adjustment for initial BMI [7]. These results show that the time and magnitude of weight gain should be taken into account when the impact of body weight on T2D incidence is assessed.

Pancreatic β-cell function starts to deteriorate from early age [20]. A study with healthy, glucose tolerant Caucasians showed that β-cell function is greatest around age 20 years and declines with age at a rate of about 1% per year [21], providing the rationale for choosing age 20 years as the baseline in our study. In contrast, insulin sensitivity was not affected by aging within the time frame studied [21].

A study demonstrated that the risk of diabetes increases with early weight gain and decreases with later weight loss [22]. Another study showed that BMI in childhood was a negative and independent predictor of insulin secretion at adulthood after adjusting for age, sex, and fat percent, indicating that pancreatic β-cell capacity may be set early in life [23]. Conceivably, rapid increase of weight could be more damaging to pancreatic β-cell function than slow increase, given the briefer period of time available to adapt to weight increase [24]. Taken together, these data suggest that rapid weight gain is more harmful to pancreatic function than slow weight gain, particularly in younger age.

The current study extends prior work by providing the detriments of weight gain on concomitant microvascular complications of T2D. In a study from the Atherosclerosis Risk in Communities, weight gainers had significantly less favorable glucose and lipid levels when compared with weight maintainers [25]. Another study showed that greater weight gain was associated with glycaemic progression in non-diabetic subjects [26]. In the present study, rapid weight gainers showed earlier diagnosis of T2D, higher level of HbA1c, and higher prevalence of microalbuminuria and diabetic retinopathy compared to relatively slow weight gainers. Although rapid weight gain may indicate other comorbidities compared to slow weight gain, these findings highlight the importance of the dynamics of weight change associated with development of T2D, glycemic control, and diabetic microvascular complications.

Several mechanisms for the weight gain and development of T2D and its complication can be postulated. Weight gain, particularly rapid increase in adiposity, leads to the alteration in gene expression of growth factors and cytokines such as transforming growth factor-β that are important in the development of diabetes and obesity-associated glomerular injury [27]. Hyperlipidemia, commonly accompanied by obesity, is a risk factor for the development of albuminuria by promoting glomerular injury through renal upregulation of sterol-regulatory element-binding proteins, which in turn induces mesangial cell proliferation and cytokine synthesis [28].

The prevalence of diabetic retinopathy was also associated with rapid and greater weight gain in our study. Obese people were 6.5 times more likely to have PDR than were those with normal weight, and the degree of obesity was positively associated with increasing severity of diabetic retinopathy [29]. These findings suggest that diabetic retinopathy is a multifactorial microvascular complication, which is associated with obesity, hyperglycemia, and blood pressure.

The MAXWEL cohort has several novel strengths. First, weight information at age 20 years was accurately obtained from official written documents in 94.5% of participants. Second, identification of diabetes was based on laboratory results, not based on self-report. Third, only newly detected subjects with diabetes were included, which enabled us to assess glycaemia and status of diabetic complications at the time of diagnosis.

The primary limitation of this study is its cross-sectional design with retrospective components: the identification of maximum weight and age at maximum weight were based on self-report. When prevalence estimates for obesity were compared, it was found that bias in self-reported weight was smaller in-person interviews than in telephone interviews [30]. In the setting of rigorous in-person interviews by physicians, it has been shown that relationships between self-reported and measured weight are strong [31]. In our sample, self-reported weight was highly accurate in randomly selected subjects. In addition, we did not assess weight fluctuation, which may affect pancreatic β-cell function [32], [33]. However, effect of weight fluctuation has not been significant after adjustment for overall weight status or attained BMI in previous studies [32], [33].

In conclusion, we found that both rapid and great weight gain were associated with not only early development of T2D and glycemic status but also microalbuminuria and diabetic retinopathy. These results support public health recommendations to reduce the risk of T2D and its microvascular complications by preventing weight gain from adolescent or early adulthood. Healthcare providers may also consider reviewing patients’ weight histories when assessing their T2D risk.

## Supporting Information

File S1 **Supporting Tables: Table A.** Variables associated with age at T2D diagnosis, HbA1c at T2D diagnosis, and urine albumin-to-creatinine ratio (U_ACR_) at T2D diagnosis in men. **Table B.** Variables associated with age at T2D diagnosis, HbA1c at T2D diagnosis, and urine albumin-to-creatinine ratio (U_ACR_) at T2D diagnosis in women. **Table C.** Variables associated with diabetic retinopathy in men. **Table D.** Variables associated with diabetic retinopathy in women.
(DOCX)

## References

[1] Shaw JE, Sicree RA, Zimmet PZ (2010) Global estimates of the prevalence of diabetes for 2010 and 2030. Diabetes Res Clin Pract 87: 4–14. 19896746 10.1016/j.diabres.2009.10.007

[2] Zhang P, Zhang X, Brown J, Vistisen D, Sicree R, et al (2010) Global healthcare expenditure on diabetes for 2010 and 2030. Diabetes Res Clin Pract 87: 293–301. 20171754 10.1016/j.diabres.2010.01.026

[3] Field AE, Coakley EH, Must A, Spadano JL, Laird N, et al (2001) Impact of overweight on the risk of developing common chronic diseases during a 10-year period. Arch Intern Med 161: 1581–6. 11434789 10.1001/archinte.161.13.1581

[4] Olefsky JM, Kolterman OG, Scarlett JA (1982) Insulin action and resistance in obesity and noninsulin-dependent type II diabetes mellitus. Am J Physiol 243: E15–E30. 7046470 10.1152/ajpendo.1982.243.1.E15

[5] Ford ES, Williamson DF, Liu S (1997) Weight change and diabetes incidence: findings from a national cohort of US adults. Am J Epidemiol 146: 214–22. 9247005 10.1093/oxfordjournals.aje.a009256

[6] Hanson RL, Narayan KM, McCance DR, Pettitt DJ, Jacobsson LT, et al (1995) Rate of weight gain, weight fluctuation, and incidence of NIDDM. Diabetes 44: 261–6. 7883111 10.2337/diab.44.3.261

[7] Wannamethee SG, Shaper AG (1999) Weight change and duration of overweight and obesity in the incidence of type 2 diabetes. Diabetes Care 22: 1266–72. 10480769 10.2337/diacare.22.8.1266

[8] Wannamethee SG, Shaper AG, Walker M (2005) Overweight and obesity and weight change in middle aged men: impact on cardiovascular disease and diabetes. J Epidemiol Community Health 59: 134–9. 15650145 10.1136/jech.2003.015651 PMC1733005

[9] Knowler WC, Barrett-Connor E, Fowler SE, Hamman RF, Lachin JM, et al (2002) Reduction in the incidence of type 2 diabetes with lifestyle intervention or metformin. N Engl J Med 346: 393–403. 11832527 10.1056/NEJMoa012512 PMC1370926

[10] Tuomilehto J, Lindstrom J, Eriksson JG, Valle TT, Hamalainen H, et al (2001) Prevention of type 2 diabetes mellitus by changes in lifestyle among subjects with impaired glucose tolerance. N Engl J Med 344: 1343–50. 11333990 10.1056/NEJM200105033441801

[11] Li G, Zhang P, Wang J, Gregg EW, Yang W, et al (2008) The long-term effect of lifestyle interventions to prevent diabetes in the China Da Qing Diabetes Prevention Study: a 20-year follow-up study. Lancet 371: 1783–9. 18502303 10.1016/S0140-6736(08)60766-7

[12] Diagnosis and classification of diabetes mellitus. Diabetes Care 35 Suppl 1S64–S71. 22187472 10.2337/dc12-s064 PMC3632174

[13] Wilkinson CP, Ferris FL III, Klein RE, Lee PP, Agardh CD, et al (2003) Proposed international clinical diabetic retinopathy and diabetic macular edema disease severity scales. Ophthalmology 110: 1677–82. 13129861 10.1016/S0161-6420(03)00475-5

[14] Resnick HE, Valsania P, Halter JB, Lin X (2000) Relation of weight gain and weight loss on subsequent diabetes risk in overweight adults. J Epidemiol Community Health 54: 596–602. 10890871 10.1136/jech.54.8.596 PMC1731720

[15] Looker HC, Knowler WC, Hanson RL (2001) Changes in BMI and weight before and after the development of type 2 diabetes. Diabetes Care 24: 1917–22. 11679457 10.2337/diacare.24.11.1917

[16] Oguma Y, Sesso HD, Paffenbarger RS Jr, Lee IM (2005) Weight change and risk of developing type 2 diabetes. Obes Res 13: 945–51. 15919849 10.1038/oby.2005.109

[17] Koh-Banerjee P, Wang Y, Hu FB, Spiegelman D, Willett WC, et al (2004) Changes in body weight and body fat distribution as risk factors for clinical diabetes in US men. Am J Epidemiol 159: 1150–9. 15191932 10.1093/aje/kwh167

[18] Brancati FL, Wang NY, Mead LA, Liang KY, Klag MJ (1999) Body weight patterns from 20 to 49 years of age and subsequent risk for diabetes mellitus: the Johns Hopkins Precursors Study. Arch Intern Med 159: 957–63. 10326937 10.1001/archinte.159.9.957

[19] McCance DR, Pettitt DJ, Hanson RL, Jacobsson LT, Bennett PH, et al (1994) Glucose, insulin concentrations and obesity in childhood and adolescence as predictors of NIDDM. Diabetologia 37: 617–23. 7926348 10.1007/BF00403382

[20] Szoke E, Shrayyef MZ, Messing S, Woerle HJ, van Haeften TW, et al (2008) Effect of aging on glucose homeostasis: accelerated deterioration of beta-cell function in individuals with impaired glucose tolerance. Diabetes Care 31: 539–43. 18083793 10.2337/dc07-1443

[21] Chiu KC, Lee NP, Cohan P, Chuang LM (2000) Beta cell function declines with age in glucose tolerant Caucasians. Clin Endocrinol (Oxf) 53: 569–75. 11106917 10.1046/j.1365-2265.2000.01132.x

[22] Black E, Holst C, Astrup A, Toubro S, Echwald S, et al (2005) Long-term influences of body-weight changes, independent of the attained weight, on risk of impaired glucose tolerance and Type 2 diabetes. Diabet Med 22: 1199–205. 16108849 10.1111/j.1464-5491.2005.01615.x

[23] Thearle MS, Bunt JC, Knowler WC, Krakoff J (2009) Childhood predictors of adult acute insulin response and insulin action. Diabetes Care 32: 938–43. 19228868 10.2337/dc08-1833 PMC2671129

[24] Lingohr MK, Buettner R, Rhodes CJ (2002) Pancreatic beta-cell growth and survival–a role in obesity-linked type 2 diabetes? Trends Mol Med 8: 375–84. 12127723 10.1016/s1471-4914(02)02377-8

[25] Truesdale KP, Stevens J, Cai J (2005) The effect of weight history on glucose and lipids: the Atherosclerosis Risk in Communities Study. Am J Epidemiol 161: 1133–43. 15937022 10.1093/aje/kwi151 PMC3234677

[26] Rhee EJ, Choi JH, Yoo SH, Bae JC, Kim WJ, et al (2011) The association of unintentional changes in weight, body composition, and homeostasis model assessment index with glycemic progression in non-diabetic healthy subjects. Diabetes Metab J 35: 138–48. 21738896 10.4093/dmj.2011.35.2.138 PMC3122898

[27] Chalmers L, Kaskel FJ, Bamgbola O (2006) The role of obesity and its bioclinical correlates in the progression of chronic kidney disease. Adv Chronic Kidney Dis 13: 352–64. 17045221 10.1053/j.ackd.2006.07.010

[28] Rutledge JC, Ng KF, Aung HH, Wilson DW (2010) Role of triglyceride-rich lipoproteins in diabetic nephropathy. Nat Rev Nephrol 6: 361–70. 20440276 10.1038/nrneph.2010.59

[29] Dirani M, Xie J, Fenwick E, Benarous R, Rees G, et al (2011) Are obesity and anthropometry risk factors for diabetic retinopathy? The diabetes management project. Invest Ophthalmol Vis Sci 52: 4416–21. 21482643 10.1167/iovs.11-7208

[30] Ezzati M, Martin H, Skjold S, Vander HS, Murray CJ (2006) Trends in national and state-level obesity in the USA after correction for self-report bias: analysis of health surveys. J R Soc Med 99: 250–7. 16672759 10.1258/jrsm.99.5.250 PMC1457748

[31] McAdams MA, van Dam RM, Hu FB (2007) Comparison of self-reported and measured BMI as correlates of disease markers in US adults. Obesity (Silver Spring) 15: 188–96. 17228047 10.1038/oby.2007.504

[32] Waring ME, Eaton CB, Lasater TM, Lapane KL (2010) Incident diabetes in relation to weight patterns during middle age. Am J Epidemiol 171: 550–6. 20110286 10.1093/aje/kwp433 PMC2842224

[33] Field AE, Manson JE, Laird N, Williamson DF, Willett WC, et al (2004) Weight cycling and the risk of developing type 2 diabetes among adult women in the United States. Obes Res 12: 267–74. 14981219 10.1038/oby.2004.34

