# Supplementary material for: The Association of Maximum Body Weight on the Development of Type 2 Diabetes and Microvascular Complications: MAXWEL Study
Source: PLoS One. 2013 Dec 4;8(12):e80525. doi: 10.1371/journal.pone.0080525 (PMC3851456; doi:10.1371/journal.pone.0080525)
Supplement: File S1 — Supporting Tables: Table A. Variables associated with age at T2D diagnosis, HbA1c at T2D diagnosis, and urine albumin-to-creatinine ratio (UACR) at T2D diagnosis in men. Table B. Variables associated with age at T2D diagnosis, HbA1c at T2D diagnosis, and urine albumin-to-creatinine ratio (UACR) at T2D diagnosis in women. Table C. Variables associated with diabetic retinopathy in men. Table D. Variables associated with diabetic retinopathy in women. (DOCX) [file pone.0080525.s001.docx]

| **Table A.** Variables associated with age at T2D diagnosis, HbA1c at T2D diagnosis, and urine albumin-to-creatinine ratio (U_ACR_) at T2D diagnosis in men | | | | | | | |
| --- | --- | --- | --- | --- | --- | --- | --- |
|  |  | | Standardized Beta | | *P** | 95% CI | |
|  |  |  |  |  |  | Lower | Upper |
| ***a. For age at T2D diagnosis***^†^ | | | | | | | |
| BMI_20y_ (kg/m^2^) | |  | | -0.07 | 0.004 | -0.38 | -0.09 |
| Alcohol (moderate or less vs. heavy) | |  | | -0.09 | 0.014 | -1.89 | -0.53 |
| Smoking (non vs. ex vs. current) | |  | | -0.13 | <0.001 | -2.28 | -1.02 |
| Exercise (regular vs. irregular vs. no) | |  | | -0.08 | 0.002 | -1.61 | -0.37 |
| Family history of diabetes (no vs. yes) | |  | | -0.12 | <0.001 | -3.42 | -1.38 |
| ΔWt (kg) | |  | | -0.23 | <0.001 | -0.37 | -0.18 |
| Rate_max_wt_ (kg/year) | |  | | -0.15 | <0.001 | -0.39 | -0.04 |
| ***b. For HbA1c at T2D diagnosis***^‡^ | | | | | | | |
| Age_T2D_ (year) | |  | | -0.12 | <0.001 | -0.03 | -0.01 |
| BMI_20y_ (kg/m^2^) | |  | | 0.09 | 0.013 | 0.01 | 0.07 |
| Smoking (non vs. ex vs. current) | |  | | 0.06 | 0.032 | 0.01 | 0.21 |
| Exercise (regular vs. irregular vs. no) | |  | | 0.11 | 0.001 | 0.09 | 0.29 |
| ΔWt (kg) | |  | | 0.12 | 0.012 | 0.01 | 0.04 |
| Rate_max_wt_ (kg/year) | |  | | 0.12 | 0.006 | 0.10 | 0.59 |
| ***c. For U_ACR_*** ^#^ ***at T2D diagnosis ^¶^*** | | | | | | | |
| BMI_20y_ (kg/m^2^) | |  | | 0.10 | 0.008 | 0.02 | 0.08 |
| SBP (mmHg) | |  | | 0.19 | <0.001 | 0.01 | 0.02 |
| HbA1c_T2D_ (%) | |  | | 0.19 | <0.001 | 0.14 | 0.26 |
| Log-triglyceride/HDL-cholesterol | |  | | 0.05 | 0.031 | 0.21 | 4.40 |
| ΔWt (kg) | |  | | 0.09 | 0.002 | 0.01 | 0.03 |
| Rate_max_wt_ (kg/year) | |  | | 0.10 | 0.001 | 0.01 | 0.04 |
| †Common covariates: sex, BMI_20y_, alcohol intake, smoking status, exercise habit, family history of diabetes, ΔWt, and Rate_max_wt._ ‡ Common covariates + Age_T2D,_ ¶ Common covariates + Age_T2D_ + HbA1c_T2D_ + log-triglyceride/HDL-cholesterol. # Log-transformed value was used. | | | | | | | |

| **Table B.** Variables associated with age at T2D diagnosis, HbA1c at T2D diagnosis, and urine albumin-to-creatinine ratio (U_ACR_) at T2D diagnosis in women | | | | | | | |
| --- | --- | --- | --- | --- | --- | --- | --- |
|  |  | | Standardized Beta | | *P** | 95% CI | |
|  |  |  |  |  |  | Lower | Upper |
| ***a. For age at T2D diagnosis***^†^ | | | | | | | |
| BMI_20y_ (kg/m^2^) | |  | | -0.11 | 0.012 | -0.59 | -0.12 |
| Alcohol (moderate or less vs. heavy) | |  | | -0.08 | 0.014 | -3.02 | -0.51 |
| Exercise (regular vs. irregular vs. no) | |  | | -0.04 | 0.048 | -1.12 | -0.34 |
| Family history of diabetes (no vs. yes) | |  | | -0.11 | <0.001 | -3.19 | -1.53 |
| ΔWt (kg) | |  | | -0.24 | <0.001 | -0.33 | -0.09 |
| Rate_max_wt_ (kg/year) | |  | | -0.17 | <0.001 | -0.35 | -0.02 |
| ***b. For HbA1c at T2D diagnosis***^‡^ | | | | | | | |
| Age_T2D_ (year) | |  | | -0.17 | <0.001 | -0.03 | -0.01 |
| BMI_20y_ (kg/m^2^) | |  | | 0.17 | <0.001 | 0.05 | 0.13 |
| Smoking (non vs. ex vs. current) | |  | | 0.10 | 0.003 | 0.12 | 0.63 |
| Exercise (regular vs. irregular vs. no) | |  | | 0.07 | 0.023 | 0.02 | 0.23 |
| ΔWt (kg) | |  | | 0.37 | <0.001 | 0.04 | 0.08 |
| Rate_max_wt_ (kg/year) | |  | | 0.06 | 0.056 | 0.00 | 0.35 |
| ***c. For U_ACR_*** ^#^ ***at T2D diagnosis ^¶^*** | | | | | | | |
| BMI_20y_ (kg/m^2^) | |  | | 0.09 | 0.012 | 0.01 | 0.06 |
| SBP (mmHg) | |  | | 0.18 | <0.001 | 0.01 | 0.03 |
| HbA1c_T2D_ (%) | |  | | 0.21 | 0.001 | 0.18 | 0.27 |
| Log-triglyceride/HDL-cholesterol | |  | | 0.07 | 0.043 | 0.16 | 4.21 |
| ΔWt (kg) | |  | | 0.12 | 0.001 | 0.02 | 0.05 |
| Rate_max_wt_ (kg/year) | |  | | 0.11 | <0.001 | 0.02 | 0.06 |
| †Common covariates: sex, BMI_20y_, alcohol intake, smoking status, exercise habit, family history of diabetes, ΔWt, and Rate_max_wt._ ‡ Common covariates + Age_T2D,_ ¶ Common covariates + Age_T2D_ + HbA1c_T2D_ + log-triglyceride/HDL-cholesterol.  # Log-transformed value was used. | | | | | | | |

| **Table C.** Variables associated with diabetic retinopathy in men^†‡^ | | | | |
| --- | --- | --- | --- | --- |
|  | OR | 95% CI | | *P** |
|  |  | Lower | Upper |  |
| BMI_20y_ (kg/m^2^) | 1.09 | 1.01 | 1.17 | 0.030 |
| SBP/DBP ≥ 140/90 mmHg or blood pressure medication | 2.13 | 1.39 | 3.27 | 0.001 |
| HbA1c_T2D_ (%) | 1.12 | 1.01 | 1.27 | 0.001 |
| ΔWt (kg) | 1.05 | 1.02 | 1.08 | 0.001 |
| Rate_max_wt_ (kg/year) | 1.13 | 1.05 | 1.22 | 0.002 |
| *Corrected P by Bonferroni method, ^†^Covariates: Age_T2D_, sex, BMI_20y_, SBP/DBP ≥ 140/90 mmHg or blood pressure medication, alcohol intake, smoking status, exercise habit, family history of diabetes, HbA1c_T2D_, log-triglyceride/HDL-cholesterol, ΔWt, and Rate_max_wt,_ ^‡^Both nonproliferative and proliferative diabetic retinopathy were combined. | | | | |

| **Table D.** Variables associated with diabetic retinopathy in women^†‡^ | | | | |
| --- | --- | --- | --- | --- |
|  | OR | 95% CI | | *P** |
|  |  | Lower | Upper |  |
| BMI_20y_ (kg/m^2^) | 1.08 | 1.02 | 1.19 | 0.024 |
| SBP/DBP ≥ 140/90 mmHg or blood pressure medication | 2.05 | 1.18 | 3.59 | 0.012 |
| HbA1c_T2D_ (%) | 1.49 | 1.28 | 1.72 | <0.001 |
| ΔWt (kg) | 1.02 | 1.01 | 1.07 | 0.001 |
| Rate_max_wt_ (kg/year) | 1.07 | 1.01 | 1.14 | 0.032 |
| *Corrected P by Bonferroni method, ^†^Covariates: Age_T2D_, sex, BMI_20y_, SBP/DBP ≥ 140/90 mmHg or blood pressure medication, alcohol intake, smoking status, exercise habit, family history of diabetes, HbA1c_T2D_, log-triglyceride/HDL-cholesterol, ΔWt, and Rate_max_wt,_ ^‡^Both nonproliferative and proliferative diabetic retinopathy were combined. | | | | |
